# Alveolar macrophage phenotype expression in airway-instilled bone marrow cells in mice

**DOI:** 10.1186/s40064-015-1525-2

**Published:** 2015-12-12

**Authors:** Masayuki Okui, Taichiro Goto, Keisuke Asakura, Ikuo Kamiyama, Takashi Ohtsuka

**Affiliations:** Division of General Thoracic Surgery, Department of Surgery, School of Medicine, Keio University, Shinjuku-ku, Tokyo, 160-8582 Japan

**Keywords:** Hematopoietic stem cells, Pneumonectomy, Bone marrow, Differentiation, Alveolar macrophage

## Abstract

No uniform consensus has been established regarding post-pneumonectomy lung regeneration. This study was undertaken to determine whether airway-instilled lung- or bone marrow-derived cells are able to differentiate and reconstitute as lung component cells in the course of post-pneumonectomy lung growth. Bone marrow cells or lung cells obtained from C57 black (BL)/6-GFP mice were intratracheally instilled into C57BL/6 mice treated with left pneumonectomy and cell differentiation was examined. It is unclear whether intratracheally instilled lung or bone marrow cells differentiate into non-hematopoietic cells after pneumonectomy. However, regardless of whether pneumonectomy is performed, intratracheally instilled bone marrow cells display a surface antigen profile that is similar to alveolar macrophages. Furthermore, these newly differentiated macrophages function similarly to resident macrophages in terms of TNF-α production, suggesting that bone marrow stem cells acquire the same macrophage phenotype. In conclusion, intratracheally instilled bone marrow cells adapt to the surrounding microenvironment, directly differentiating into alveolar macrophages, and remain in the alveolar space for at least 3 months.

## Background

Lung resection continues to be the primary treatment for lung cancer. One of the most important factors that determine the resectability level is the residual lung function. It is well known clinically that after lung resection in adults the residual lung increases in volume to some extent, but this is considered to be primarily due to hyperinflation, with minimal recovery (Laros and Westermann 1987). In children, recovery of lung function after lung resection has been reported (Nakajima et al. 1998). The fact that alveoli multiply after birth up until about 8 years of age is well established (Kreisel et al. 2004). In addition, adult lungs transplanted into immature recipients have been reported to show hyperplastic growth (Binns et al. 1997). These results taken together suggest that restoration or augmentation of compensatory growth capability is possible, at least in part, even in the adult lungs.

Compensatory lung growth after lung resection has been reported in many animal models (Jancelewicz et al. 2010; Takahashi et al. 2011; Wada et al. 2012; Fehrenbach et al. 2008; Takahashi et al. 2010; Voswinckel et al. 2004). It is well described as a phenomenon, yet little is known about its nature, extent or capacity for modulation. Moreover, whether regeneration of new lung tissue is involved in the process of compensatory lung growth is at present controversial.

The objective of our study was to determine whether airway-instilled lung- or bone marrow-derived cells can differentiate and reconstitute lung component cells in post-pneumonectomy lung growth. To directly investigate this, GFP-positive lung or bone marrow cells were instilled into the airway without isolating and conditioning the stem cells prior to instillation, and flow cytometry was performed to quantitate the fraction of the lung component cells that was replaced by GFP-positive cells. Various kinds of cells are supposed to work together in lung reconstitution, so the experimental step of stem cell isolation might exclude or otherwise result in a malfunction of certain key stem cells. Thus, in order to maximize the chances of lung reconstitution, our method is the first, to the best of our knowledge, to skip any isolation and conditioning of the stem cells before instilling them into the airways. We investigated the mechanism by which mesenchymal and bone marrow stem cells are in their naïve state involved in the reconstitution of the lung. In addition, we also examined whether intratracheally instilled bone marrow hematopoietic cells would adapt to the microenvironment in the airway and assume proper patterns of differentiation. Moreover, the function and life span of these cells in the lung were also investigated.

## Methods

### Reagents

The reagents and materials used for this study were obtained from the following sources: dispase II was from Roche Applied Science (Indianapolis, IN, USA); fetal calf serum and collagenase type 1 were from Invitrogen (Carlsbad, CA, USA); phosphate buffered saline (PBS) and paraformaldehyde were from Wako (Osaka, Japan); deoxyribonuclease I from bovine pancreas and red blood cell lysing buffer were from Sigma-Aldrich (St. Louis, MO, USA); brefeldin A and permealization buffer were from eBioscience (San Diego, CA, USA) and 7-AAD solution and BD Fc Block were from BD Pharmingen (San Diego, CA, USA). The anti-mouse antibodies used for flow cytometry included the following: CD45 (clone: 30-F11; eFluor 450), CD105 (clone: MJ7/18; PE), E-cadherin (clone: DECMA-1; eFluor 660), F4/80 (clone: BM8; eFluor 450), CD11c (clone: N418; PE), CD11b (clone: M1/70; PE-Cy7, eBioscience, San Diego, CA, USA); TNF-α (clone: MP6-XT22; PE-Cy7), VE-cadherin (clone: 11D4.1; Alexa Fluor 647, BD Pharmingen, San Diego, CA, USA); Ep-CAM (clone: G8.8; PE), Ly6G (clone: 1A8; APC-Cy 7, BioLegend, San Diego, CA, USA). The anti-mouse antibodies used for immunofluorescence included the following: GFP (ab290, AbCAM, Cambridge, MA, USA), F4/80 (AbD Serotec, Oxford, UK), and MOMA-2 (AbD Serotec). Donkey anti-rat IgG and goat anti-rabbit IgG (Jackson ImmunoResearch Laboratories, West Grove, PA, USA) were used as secondary antibodies for double immunofluorescence staining.

### Mice

Adult male C57BL/6 and transgenic mice expressing GFP, ubiquitously under control of the chicken b-actin promoter with the cytomegalovirus enhancer (C57BL/6-Tg[CAG-EGFP]), were purchased from Japan SLC (Shizuoka, Japan). The ages of the mice ranged from 6 to 8 weeks. The mice were maintained in a full barrier facility until experimental use, and all of the experiments received institutional approval.

### Experimental groups

According to the presence or absence of the GFP fluorescence of the intratracheally instilled lung or bone marrow cells and whether the recipient C57BL/6 mice were treated with left pneumonectomy, the mice were divided into experimental groups A, B and C. The recipient mice were randomly assigned to one of the three experimental groups. Group A consisted of C57BL/6 mice that were treated with left pneumonectomy and then received intratracheal instillation of lung cells or bone marrow cells obtained from wild-type mice. Group B consisted of untreated C57BL/6 mice that received intratracheal instillation of lung cells or bone marrow cells obtained from GFP mice. Group C consisted of C57BL/6 mice that were treated with left pneumonectomy and then received intratracheal instillation of lung cells or bone marrow cells obtained from GFP mice.

### Pneumonectomy

In the pneumonectomy group, the mice were anesthetized with 100 mg/kg of ketamine and 10 mg/kg of xylazine administered subcutaneously. They were intubated with a 20-gauge catheter and connected to a rodent ventilator, adjusted to maintain a respiratory rate of 120 breaths/min, a tidal volume of 10 mL/kg, 2-cm H_2_O-positive end-expiratory pressure, and 0.21 inspired oxygen. A 20-mm-long posterolateral skin incision was made, followed by thoracotomy in the fifth intercostal space along with dissection of the serratus anterior and latissimus dorsi muscles. The left main bronchus with the left pulmonary artery and vein were clipped at the hilum using a surgical stapler before removal of the lung. The whole left lung was resected from the pleural cavity. The tidal volume was reduced from 10 to 6 mL/kg after the lung was removed. The fifth intercostal space was closed with a single surgical suture, and the skin and muscle incisions were closed with two sutures to avoid excessive tension on the muscles. The duration of the mechanical ventilation for the entire surgical procedure was approximately 10 min. All of the mice recovered quickly after the termination of mechanical ventilation and were promptly extubated. The mice were observed daily for any signs of distress or changes in behavior.

### Isolation of single lung cells for the airway instillation

The mice received an overdose of inhaled isoflurane and their pulmonary vasculature was perfused with PBS via the right ventricle. The PBS-perfused lungs were isolated, dispase II solution was infused into the lungs via the trachea and the trachea was then ligated with silk suture. After incubation for 45 min at 37 °C, the lungs were separated from the other mediastinal organs. Each lung was minced and digested in PBS with 0.1 % collagenase, 0.01 % deoxiribonuclease I and 5 mM CaCl_2_ for 10 min (37 °C). Cells were suspended in red blood cell lysing buffer to remove red blood cells, washed once with PBS, centrifuged and suspended in PBS.

### Isolation of bone marrow cells for the airway instillation

Bone marrow cells were collected from the femurs of the donor mice by aspiration and flushing, and were then filtered through a 40-mm cell strainer (BD Biosciences, San Jose, CA, USA).

### Intratracheal instillation

Intratracheal instillation was performed in the C57BL/6 mice at 24 h after pneumonectomy and also in the untreated C57BL/6 mice. After inducing anesthesia with ketamine and xylazine, the trachea was surgically exposed, and a total volume of 50 μL of the lung or bone marrow cell suspensions was instilled via an angiocatheter through the trachea. In each recipient mouse, 2.03 × 10^6^ ± 0.08 × 10^6^ lung cells or 4.35 × 10^6^ ± 2.02 × 10^6^ bone marrow cells were instilled respectively.

### Isolation of single lung cells for flow cytometry

At 30 days after instillation, the mice received an overdose of inhaled isoflurane, and single lung cell suspensions were prepared from the remaining lungs by a method similar to the one previously described. This digestion protocol does not result in the loss of the antigenicity of the hematopoietic, endothelial or epithelial cell markers used for flow cytometry.

### Flow cytometry of single-lung cell preparations

Single lung cells were suspended in 2 % fetal calf serum/PBS. BD Fc Block was added to block Fc receptors, and after 5 min of incubation (4 °C), fluorochrome-conjugated antibodies were added to the cell suspension and incubated for 30 min (4 °C). The cells were then washed, counterstained with 7-AAD (BD Biosciences Pharmingen), suspended in PBS and then analyzed using a Gallios flow cytometer (Beckman Coulter, Inc.).

Doublets were excluded through FSC-A/FSC-H. Data were collected by gating for 7-AAD-negative cells. Gates of interest for each fluorochrome were drawn using control antibodies of the same isotype. For each study, all of the specific antibodies were performed in all of the combinations to compensate for the overlapping fluorescence of one fluorochrome into the channel of others. Data were analyzed using the Flow Jo software (Treestar, San Carlos, CA, USA).

### Bronchoalveolar lavage

Three months after the GFP-positive lung or bone marrow cells were intratracheally instilled into the untreated C57BL/6 mice, BAL was performed in the recipient mice. BAL cells were collected by lavaging the whole lungs with 5.0 mL of PBS in 1.0 mL aliquots via tracheal cannula and slow withdrawal. The BAL cells were stained with fluorochrome-conjugated antibodies and analyzed by flow cytometry.

### Immunofluorescence

For immunofluorescence, the BAL cells were affixed to slides using cytospin. They were then fixed with 4 % paraformaldehyde, and incubated in 5 % BSA and 0.1 % Triton X-100 to block nonspecific binding and permeabilize the cell membrane, respectively. The cells were then stained with primary and secondary antibodies according to standard procedures. Negative controls were performed with immunoglobulin G from the species from which the individual primary antibodies were obtained. Slides were mounted with Vectashield containing DAPI (Vector Laboratories, Burlingame, CA, USA) and visualized under confocal microscopy (LSM710 ZEN, Zeiss). Stacks of images were obtained in the z-direction throughout the cells, and composite images were used for comparison.

### Intracellular staining for TNF-α

Three months after the GFP-positive bone marrow cells were intratracheally instilled into the untreated C57BL/6 mice, BAL cells were obtained. Then these cells were incubated with or without 10 ng/ml LPS in medium (10 % FCS–RPMI 1640) containing brefeldin A (3.0 μg/ml) for 2 h at 37 °C. Cells were centrifuged and resuspended with 2 % FCS–PBS. To prevent nonspecific binding of antibody to Fc receptors, cells were incubated with a rat anti-mouse CD16/CD32 antibody. Staining for the cell surface antigens Ly-6G and F4/80 was performed with fluorochrome-conjugated antibodies. Cells were washed and fixed with 2 % paraformaldehyde. Cells were washed again and suspended with permealization buffer. Cells were then incubated with a PE-Cy7-conjugated anti-TNF-α antibody or isotype control antibody. Cells were washed and resuspended for flow cytometric analysis. In this study, the macrophages were identified as F4/80^+^ Ly-6G^−^ cells.

## Results

### Flow cytometric analysis of single-lung cell suspensions

**Fig. 1:**
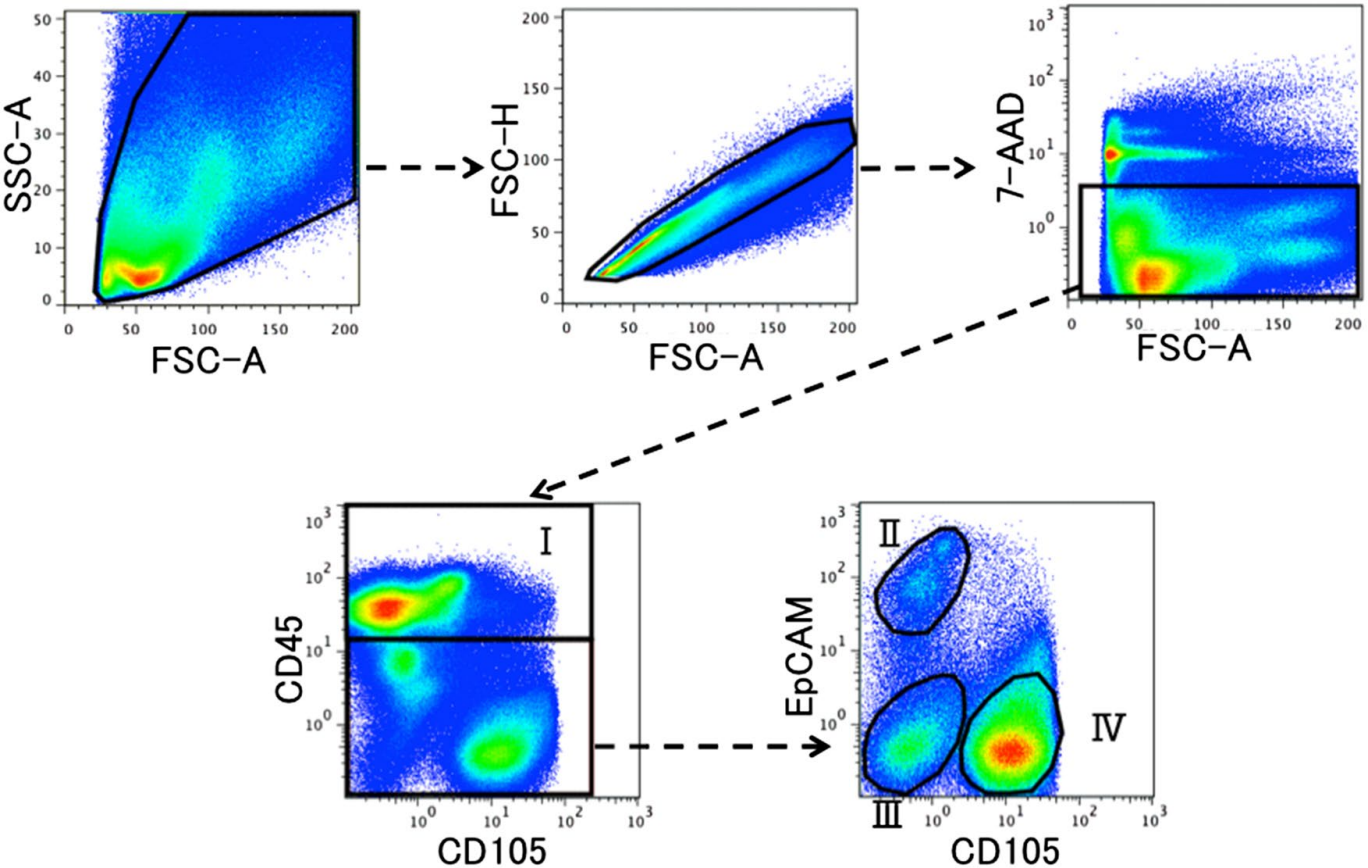
Protocol of the flow cytometric analysis. Single lung cells were obtained from the mice and analyzed by flow cytometry. Most of the single lung cells, distributed according to their values of forward and side scatter, were gated at the initial window (the *upper left panel*). Next, doublets and dead cells were excluded by FSC-A/FSC-W and 7-AAD staining, respectively (the upper middle and upper right panels). The expression of the pan-leukocyte marker CD45 designated a hematopoietic fragment (fragment I). CD45-negative cells were further subjected to flow cytometric analysis for the expression of the endothelial cell marker CD105 and epithelial marker EpCAM. The cells were divided into the following fragments: the EpCAM^+^ CD105^−^ fragment (fragment II: epithelial cells), EpCAM^−^ CD105^−^ fragment (fragment III: mesenchymal cells) and EpCAM^−^ CD105^+^ fragment (fragment IV: endothelial cells). We classified single lung cells into the fragments I to IV according to this protocol and analyzed the cells for the presence or absence of GFP fluorescence in each fragment in subsequent experiments

**Fig. 2:**
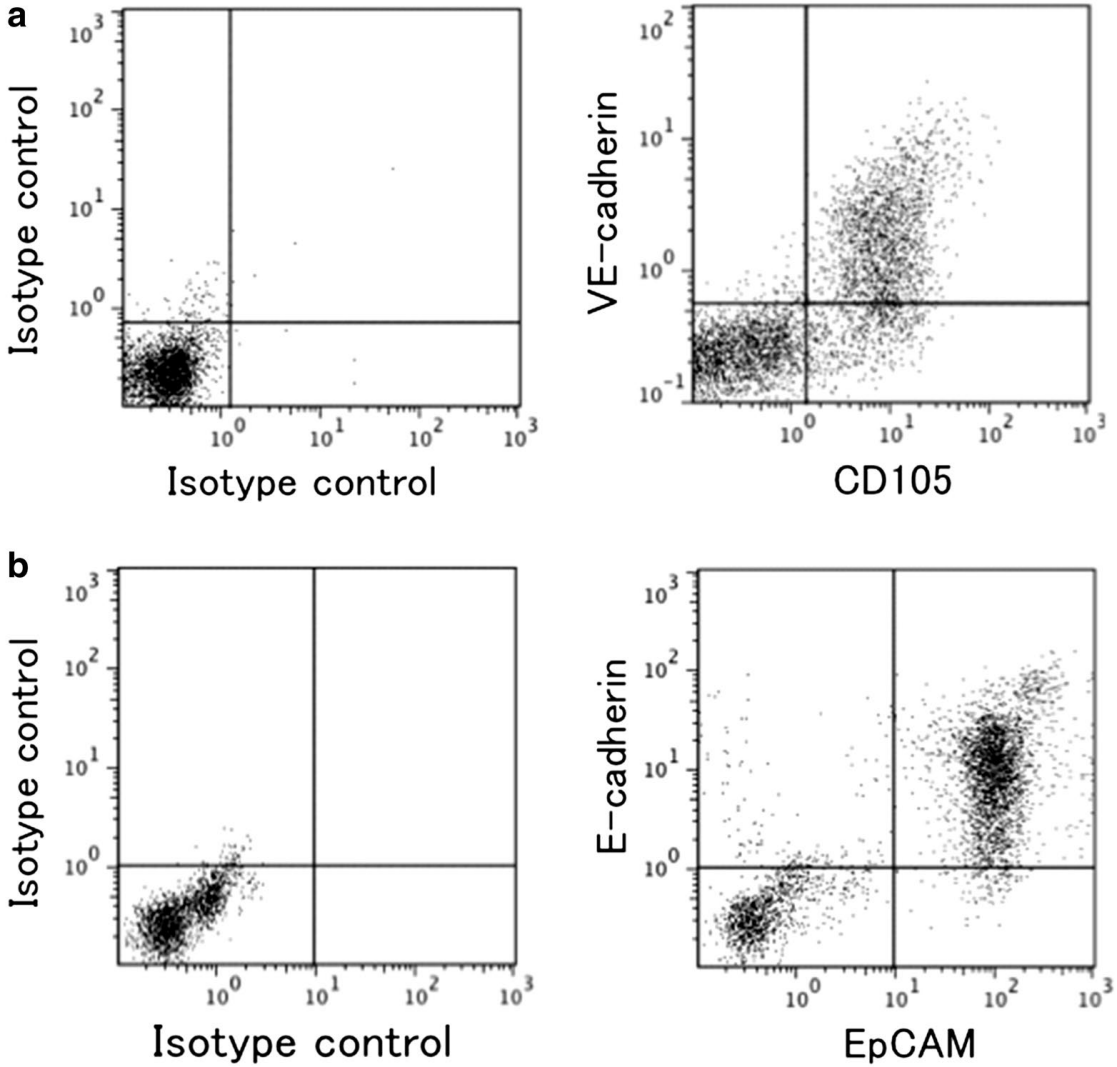
Specificity of the endothelial and epithelial markers. Single lung cells were analyzed for the endothelial markers CD105 and VE-cadherin as well as the epithelial markers EpCAM and E-cadherin. **a** Most cells that expressed one endothelial marker expressed both (n = 3, with similar results). **b** Most cells that expressed one epithelial marker also expressed both (n = 3, with similar results)

Single lung cells were isolated from the mice and subjected to flow cytometric analysis for CD45, EpCAM and CD105/endoglin as specific markers for pan-leukocytes, epithelial cells and endothelial cells, respectively. According to the flow cytometric analysis results, we classified lung component cells into hematopoietic (CD45^+^ cells), epithelial (CD45^−^, EpCAM^+^ and CD105^−^ cells), mesenchymal (CD45^−^, EpCAM^−^ and CD105^−^ cells) and endothelial cell fragments (CD45^−^, EpCAM^−^ and CD105^+^ cells; Fig. 1). Most cells that expressed one endothelial cell marker (CD105 or VE-cadherin) expressed both (Fig. 2a), and most cells that expressed one epithelial marker (EpCAM or E-cadherin) expressed both (Fig. 2b). Thus, each antibody was found to be endothelial- or epithelial-specific.

### GFP expression in lungs subjected to lung cell instillation

**Fig. 3:**
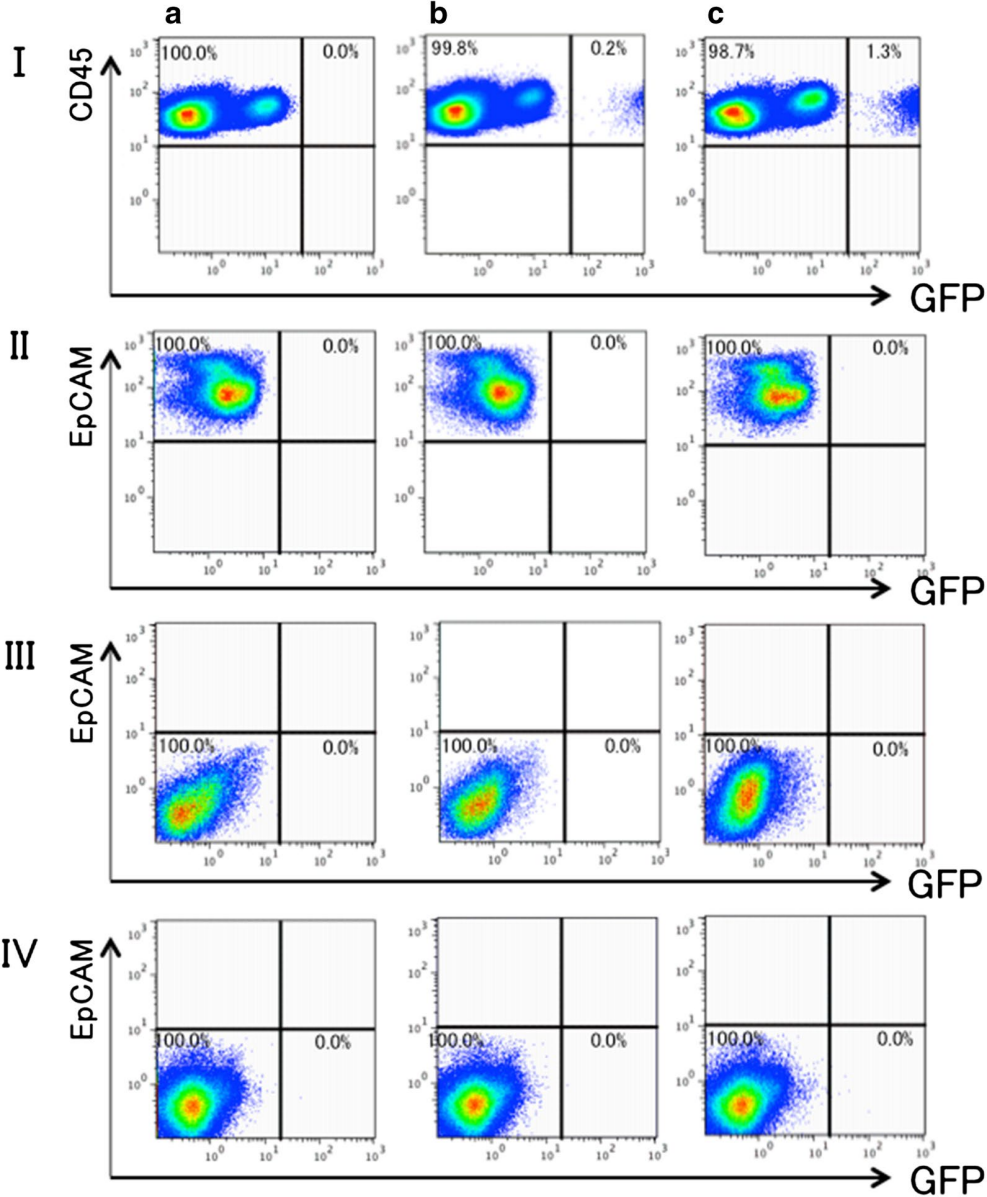
Expression of GFP in the lung subjected to intratracheal instillation of GFP-expressing lung cells. Lung single-cell digests were obtained from the recipient mice at 1 month after instillation of the lung cell suspensions. Group A was the negative control for GFP fluorescence. Regardless of the group to which they belonged, no GFP-positive cell was detected in any of the epithelial, mesenchymal and endothelial cell fragments. However, the hematopoietic cell fragments contained GFP-positive cell populations in groups B and C. The experiment was repeated 3 times (n = 3), yielding similar results

The mice were divided into Groups A, B and C, as described in the “Methods” section. At 1 month after intratracheal instillation, single-lung cell suspensions were prepared from the mice and subjected to flow cytometric analysis for the presence or absence of GFP-positive cells in the hematopoietic, epithelial, mesenchymal and endothelial cell fragments. Regardless of the (groups) group to which they belonged, no GFP-positive cell was detected in any of the epithelial, mesenchymal or endothelial cell fragments (Fig. 3). However, the hematopoietic cell fragments contained GFP-positive cell populations in groups B and C (Fig. 3).

### GFP expression in the lungs subjected to bone marrow cell instillation

**Fig. 4:**
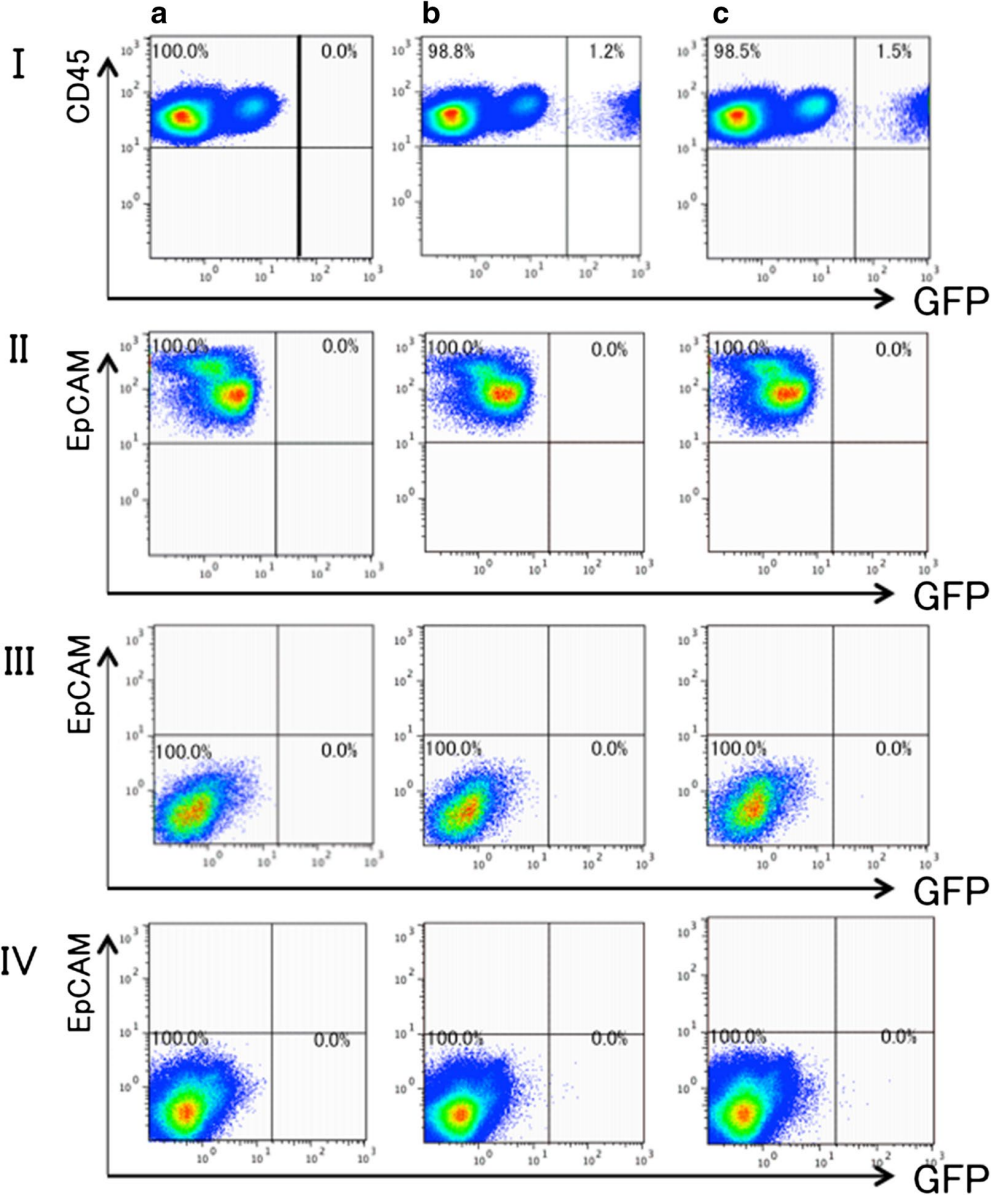
Expression of GFP in the lung subjected to intratracheal instillation of GFP-expressing bone marrow cells. Lung single-cell digests were obtained from the recipient mice at 1 month after instillation of the bone marrow cell suspensions. Group A was the negative control for GFP fluorescence. No GFP-positive cell was detected in any of the epithelial, mesenchymal or endothelial cell fragments. However, the hematopoietic cell fragments contained GFP-positive cell populations in groups B and C. The experiment was repeated 3 times (n = 3), yielding similar results

The method is quite similar to that described in “GFP expression in lungs subjected to lung cell instillation”, except that bone marrow cells were instilled into the airway instead of lung cells. The mice were divided into Group A, B and C, as described in the “Methods” section. No GFP-positive cell was detected in any of the epithelial, mesenchymal or endothelial cell fragments (Fig. 4). However, the hematopoietic cell fragments contained GFP-positive cell populations in groups B and C (Fig. 4).

### Identification of GFP-positive cells by surface antigens

**Fig. 5:**
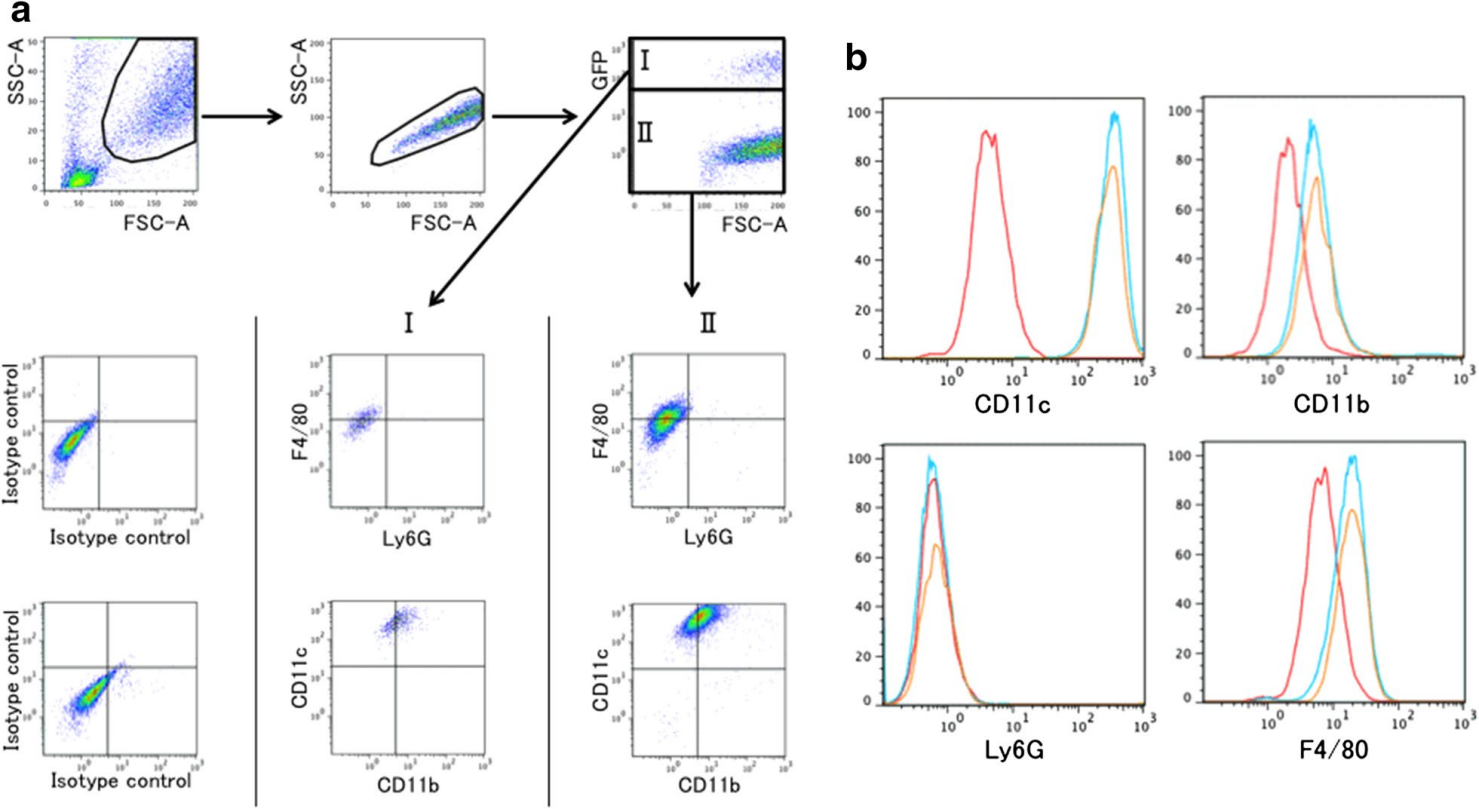
Identification of GFP-positive cells in the lung subjected to intratracheal instillation of GFP-expressing lung cells. At 3 months after intratracheal instillation of the lung component cells, bronchoalveolar lavage was performed in the recipient mice, and flow cytometric analysis of the surface antigens was performed on the BAL cells. **a** After the exclusion of doublets and dead cells, the BAL fluid cells were classified as GFP-positive cells (group I) or GFP-negative cells (group II). The cells in group II are assumed to be host-specific residential macrophages that were originally present in the pulmonary alveoli. Groups I and II were F4/80+, Ly6G−, CD11c+, and weakly CD11b+. **b** The histogram shows that the expression patterns of F4/80, Ly6G, CD11c, and CD11b were identical in groups I and II. The *red lines* indicate the isotype control; the *blue lines*, GFP-negative cells; and the *yellow lines*, GFP-positive cells. The experiment was repeated 3 times (n = 3), yielding similar results

Because the intratracheally instilled GFP-positive lung cells were positive for CD45 in the single-lung cell suspensions and did not differentiate into any other lung component cell, the instilled cells were speculated to reside in the airway. At 3 months after intratracheal instillation, the recipient mice were sacrificed and BAL cells collected. Flow cytometric analysis of surface antigen expression in the BAL cells was performed to identify GFP-positive cells. When live cells were gated, the BAL fluid cells were classified as GFP-positive or GFP-negative cells (Fig. 5a). Both the GFP-positive and GFP-negative cells were F4/80+, Ly6G−, CD11c+ and weakly CD11b+, while the GFP-positive cells exhibited a staining pattern identical to that of the residential macrophages in the GFP-negative cells (Fig. 5a, b).

**Fig. 6:**
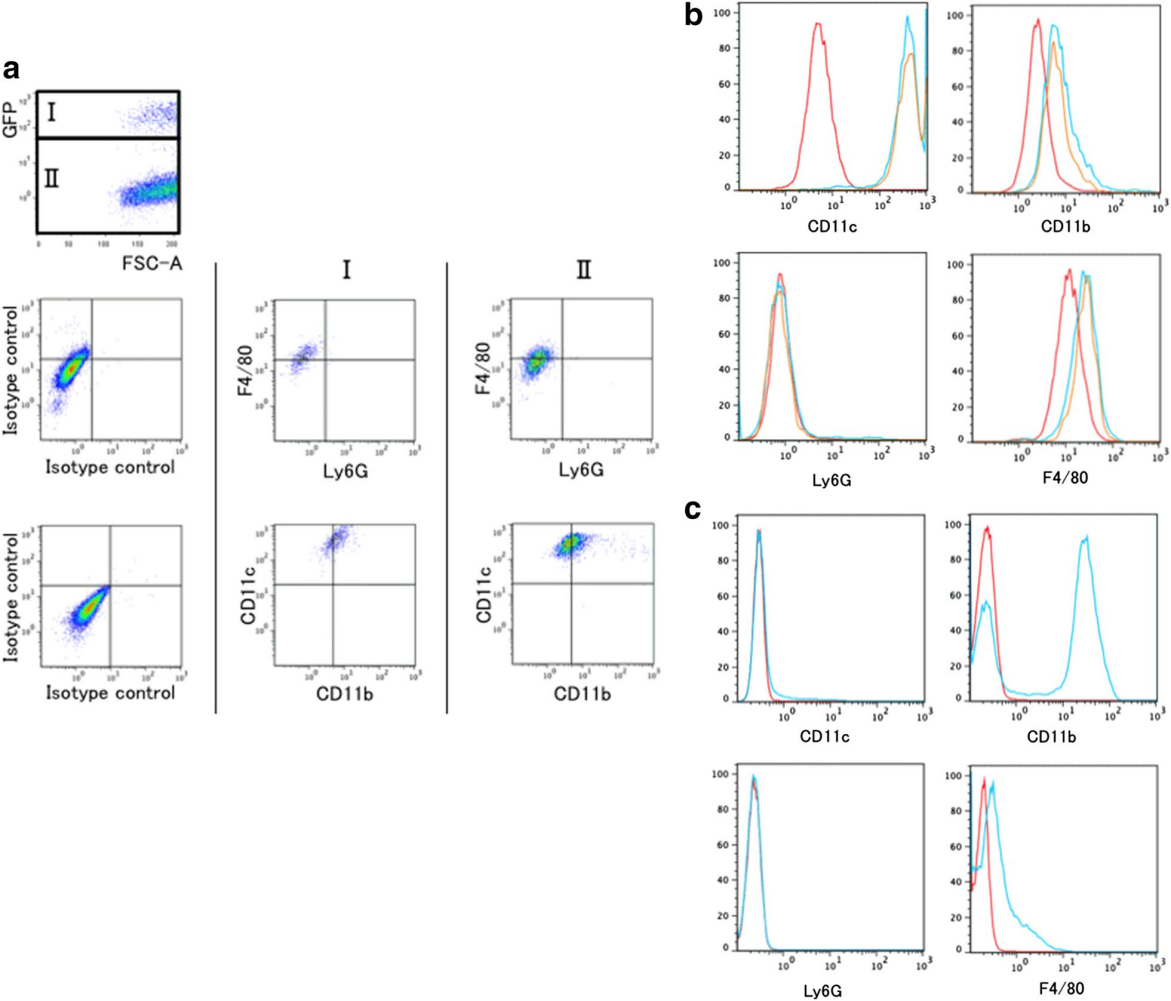
Identification of GFP-positive cells in the lung subjected to intratracheal instillation of GFP-expressing bone marrow cells. At 3 months after the intratracheal instillation of the bone marrow cells, bronchoalveolar lavage (BAL) was performed in the recipient mice, and flow cytometric analysis of surface antigens was performed on BAL cells. **a** The BAL fluid cells were classified as GFP-positive cells (group I) or GFP-negative cells (group II). Both GFP-positive BAL cells (group I) and GFP-negative BAL cells (group II) were F4/80+, Ly6G−, CD11c+, and weakly CD11b+. **b** The histogram shows that the expression patterns of F4/80, Ly6G, CD11c, and CD11b were identical in groups I and II. The *red lines* indicate the isotype control; the *blue lines*, the GFP-negative cells; and the yellow lines, GFP-positive cells (n = 3, with similar results). **c** The flow cytometric analysis of the bone marrow cells derived from the GFP mice revealed the staining patterns of CD11c, CD11b, Ly6g and F4/80 to be markedly different from those of the GFP-positive cells in the BAL cells. The *red* *lines* indicate the isotype control (n = 3, with similar results)

Next the mice that were intratracheally instilled with GFP bone marrow cells were also examined in a similar manner. Both the GFP-positive and GFP-negative cells in the BAL fluid cells were F4/80+, Ly6G−, CD11c+ and weakly CD11b+, while the GFP-positive cells exhibited a staining pattern identical to that of the GFP-negative cells (Fig. 6a, b). Furthermore, the flow cytometric analysis of the bone marrow cells derived from GFP mice revealed that the staining patterns of CD11c, CD11b, Ly6G and F4/80 markedly differed from those of the GFP-positive cells collected by BAL (Fig. 6c).

### Immunofluorescence of airway-instilled bone marrow cells

**Fig. 7:**
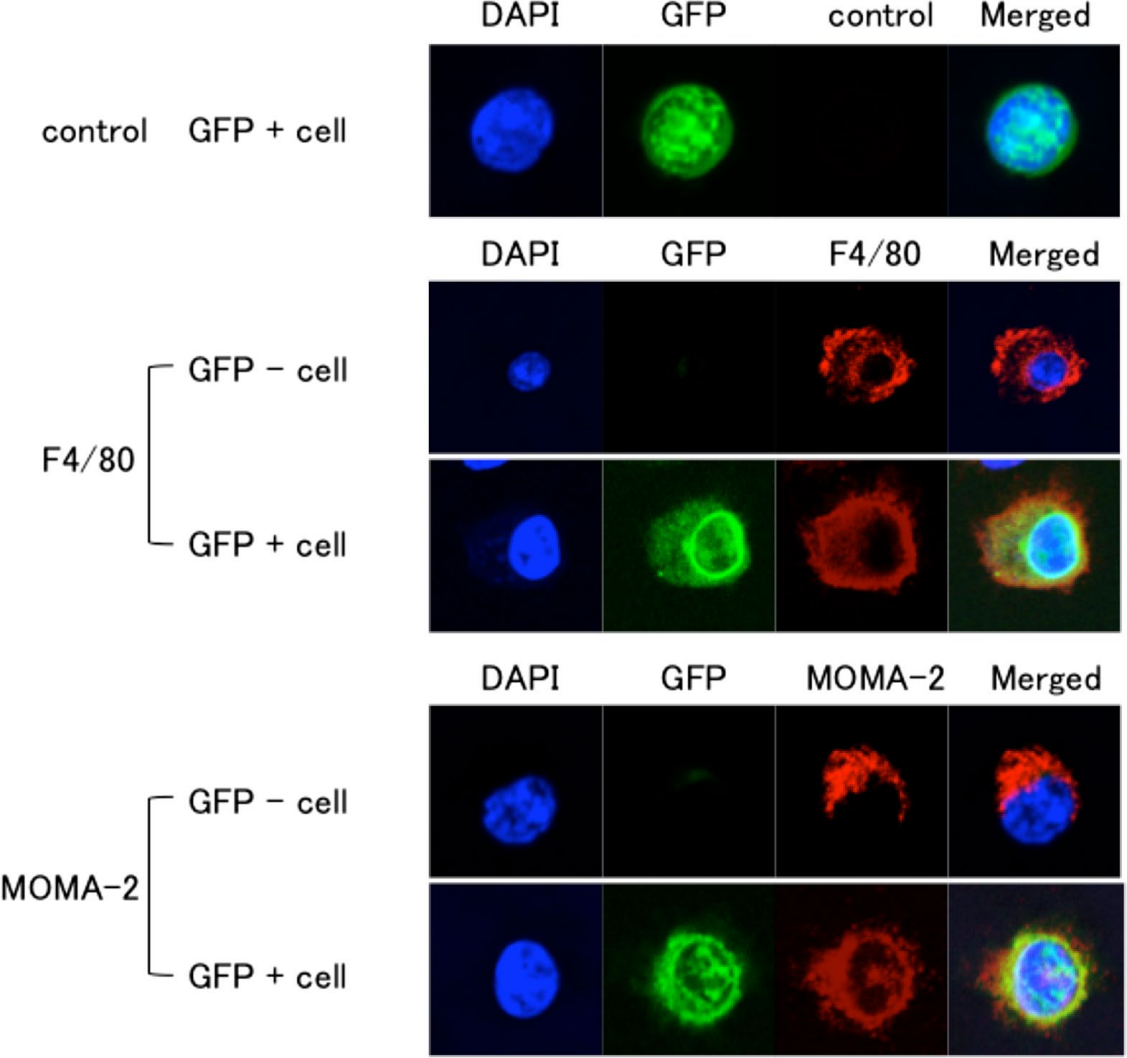
Immunofluorescence of GFP-positive cells in the airway. When BAL cells were collected and examined under confocal microscopy at 3 months after intratracheal instillation of GFP-positive bone marrow cells, the GFP-positive cells expressed both F4/80 and MOMA-2, as was also the case for the GFP-negative residential macrophages. The experiment was repeated 3 times (n = 3), yielding similar results. Typical images are shown

When BAL cells were collected and examined under confocal microscopy at 3 months after the intratracheal instillation of GFP-positive bone marrow cells, the GFP-positive cells expressed F4/80 and MOMA-2, which are markers of mature macrophages, as in the case of the GFP-negative residential macrophages (Fig. 7).

### TNF-α production in airway-instilled bone marrow cells

**Fig. 8:**
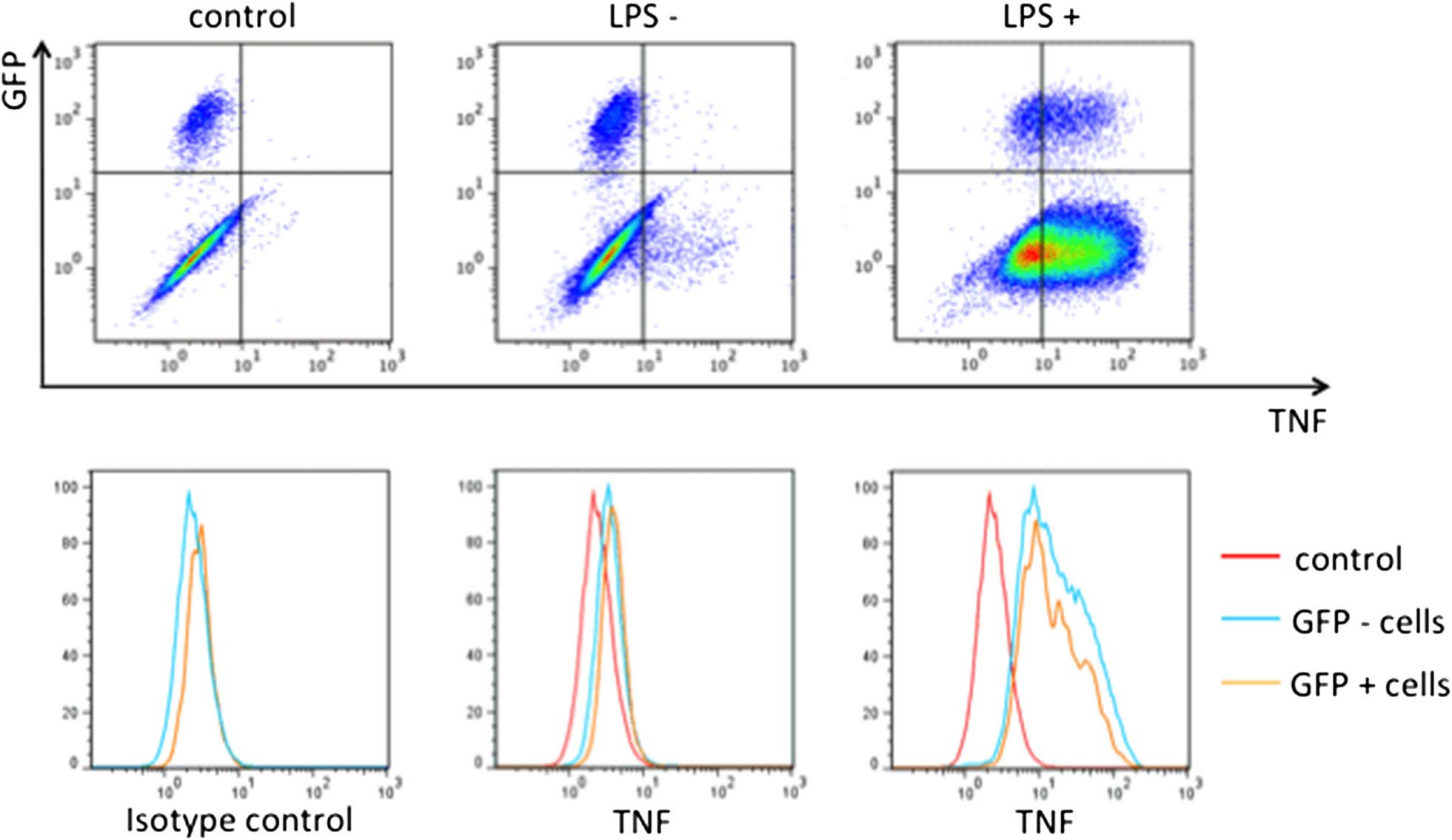
The TNF-α production by airway-instilled bone marrow cells with or without LPS treatment. Without LPS treatment, TNF-α was hardly expressed in either the GFP positive or negative cells. After 2 h of LPS treatment, intracellular staining of TNF-α revealed a quite higher level of expression in both GFP positive and negative cells compared to LPS-untreated cells. There were no differences in TNF-α expression between the GFP positive and negative cells. The experiment was repeated 3 times (n = 3), yielding similar results

In the absence of LPS treatment, TNF-α was hardly expressed in either GFP positive or negative cells (Fig. 8). After 2 h of LPS treatment, intracellular staining of TNF-α exhibited an evidently higher expression in both GFP positive and negative cells compared to LPS-untreated cells. There were no differences in TNF-α expression between GFP positive and negative cells irrespective of the presence or absence of LPS treatment.

## Discussion

The intratracheally instilled bone marrow cells did not differentiate into non-hematopoietic cells in the lung. This is consistent with the conventional concept that transdifferentiation is unlikely to occur beyond the germ layer (Nicol-Benoit et al. 2013; Terada et al. 2002; Ying et al. 2002). However, bone marrow cells exhibited similar patterns of surface antigens and similar function of TNF-α production in response to LPS treatment, with residential macrophages, suggesting hematopoietic stem cells progressed to the alveolar macrophage phenotype. In general, the process of differentiation into alveolar macrophages consists of two stages. (van Furth et al. 1972). Hematopoietic stem cells in the bone marrow first differentiate into monocytes, which are released into blood; then, the monocytes are transported through the interstitial tissue into the pulmonary alveoli, where the monocytes further differentiate into alveolar macrophages. To the best of our knowledge this is the first demonstration that bone marrow stem cells are able to adapt to the surrounding microenvironment and differentiate into alveolar macrophages in a single stage.

In terms of the limitations of this study, macrophages in the bone marrow may have proliferated in the alveoli. However, another analysis did not reveal any significant difference between the GFP-positive and GFP-negative macrophages in ki-67 staining at 1, 3, and 5 weeks after instillation (unpublished data). Thus, rapid proliferation after intratracheal instillation was unlikely for the small number of macrophages that were present in the bone marrow.

Much remains unknown about the differentiation and generation of alveolar macrophages, as well as the trigger for differentiation from monocytes into macrophages. Many basic studies have been conducted in the following manner: hematopoietic stem cells are collected and ex vivo cultured with various inducers of differentiation to observe morphological or phenotypic changes (Gupta et al. 2014; Huang et al. 2010; Lutz et al. 1999; Wang et al. 2014). Our findings in the present study enabled direct in vivo analysis of the induction of differentiation into macrophages. The in vivo generation of macrophages from hematopoietic precursors in large quantities will prove critical to the investigation of their biology.

This study shows that alveolar macrophages could be induced to incrementally proliferate in vivo for a long period. In the future, this finding may be clinically applied to achieve antitumor effects on lung malignancy or preventive effects on lung infection. Basic studies on lung cancer treatment or pneumonia prevention using animal models are awaited.

## Conclusions

In conclusion, we suggest that intratracheally instilled lung or bone marrow cells might not differentiate into non-hematopoietic cells after pneumonectomy. Meanwhile, regardless of whether pneumonectomy is performed, intratracheally instilled bone marrow cells do adapt to the surrounding microenvironment, directly differentiating into alveolar macrophages and remaining in the alveolar space for at least 3 months.
